# Supplementary material for: The influence of culture on open defaecation in some basic schools in selected districts in Ghana: A preliminary study
Source: PLoS One. 2020 Oct 6;15(10):e0239491. doi: 10.1371/journal.pone.0239491 (PMC7537866; doi:10.1371/journal.pone.0239491)
Supplement: Attachment — Submitted filename: PONE-D-20-14602_reviewer _ Reviewed.pdf [file pone.0239491.s003.pdf]

PLOS ONE

The Influence of Culture on Open Defecation in Selected Basic Schools in Ghana

--Manuscript Draft--

**Manuscript Number:**	PONE-D-20-14602
**Article Type:**	Research Article
**Full Title:**	The Influence of Culture on Open Defecation in Selected Basic Schools in Ghana
**Short Title:**	Culture and open defecation in basic schools, Ghana
**Corresponding Author:**	Dzidzo Regina Yirenya-Tawiah, PhD<br>University of Ghana<br>Legon, Accra, Greater Accra Region GHANA
**Keywords:**	Sanitation; open defecation; Behavior; education; sustainable development goals; Ghana.
**Abstract:**	Open defaecation remains major public and environmental health concerns and has attracted global attention. This study explored cultural drivers influencing open defaecation behaviour among basic school pupils in the Eastern and Volta Regions all within Southern Ghana. Self-reported data were collected from 400 pupils using structured questionnaire and focus group discussions held. The results of the study indicated that taboos, superstitions and traditional norms in their pooled state explained 63 and 23 percent of variance in pupils’ open defaecation intention and actual open defaecation behaviour respectively. In their individual state, these cultural variables yielded statistically significant effect sizes with pupils’ open defaecation behaviours: taboos: $r = 0.462$, $p<0.05$; traditional norms: $r = 0.425$, $p<0.05$; and superstition: $r = 0.728$, $p< 0.05$). Superstitions therefore constituted the key cultural driver influencing pupils’ open defaecation behaviours. It is recommended that Ministry of Education should integrate the local cultural beliefs systems relating to sanitation practices into the basic school curriculum and ensure that science teaching help pupils give scientific explanations to these cultural beliefs systems that downplay best sanitary practices in the schools. The Ministries of Education and Sanitation and Water Resources should incorporate OD issues into the educational curriculum and develop cultural sensitive educational programs for a massive educational campaign to stop OD in the schools and society at large.
**Order of Authors:**	Dzidzo Regina Yirenya-Tawiah, PhD
	Saviour Adjibolosoo
	Benjamin Ofori
	Stephen Afranie
	Philip Adongo
**Additional Information:**	
**Question**	**Response**
**Financial Disclosure**<br><br>Enter a financial disclosure statement that describes the sources of funding for the work included in this submission. Review the submission guidelines for detailed requirements. View published research articles from *PLOS ONE* for specific examples.<br><br>This statement is required for submission and **will appear in the published article** if	Research was funded through Carnegie Corporation of New York on the project “Next Generation of Academics in Africa: Enhancing the University of Ghana's Capacity to Deliver Post-graduate Research and Training.”

the submission is accepted. Please make sure it is accurate.<br><br>**Unfunded studies**<br>Enter: *The author(s) received no specific funding for this work.*<br><br>**Funded studies**<br>Enter a statement with the following details:<br>• Initials of the authors who received each award<br>• Grant numbers awarded to each author<br>• The full name of each funder<br>• URL of each funder website<br>• Did the sponsors or funders play any role in the study design, data collection and analysis, decision to publish, or preparation of the manuscript?<br>• **NO** - Include this sentence at the end of your statement: *The funders had no role in study design, data collection and analysis, decision to publish, or preparation of the manuscript.*<br>• **YES** - Specify the role(s) played.<br><br>* typeset	
**Competing Interests**<br><br>Use the instructions below to enter a competing interest statement for this submission. On behalf of all authors, disclose any competing interests that could be perceived to bias this work—acknowledging all financial support and any other relevant financial or non-financial competing interests.<br><br>This statement **will appear in the published article** if the submission is accepted. Please make sure it is accurate. View published research articles from *PLOS ONE* for specific examples.	There is no competing interest

Powered by Editorial Manager® and ProduXion Manager® from Aries Systems Corporation

**NO authors have competing interests**<br><br>Enter: *The authors have declared that no competing interests exist.*<br><br>**Authors with competing interests**<br><br>Enter competing interest details beginning with this statement:<br><br>*I have read the journal's policy and the authors of this manuscript have the following competing interests: [insert competing interests here]*<br><br>* typeset	
**Ethics Statement**<br><br>Enter an ethics statement for this submission. This statement is required if the study involved:<br><br>• Human participants<br>• Human specimens or tissue<br>• Vertebrate animals or cephalopods<br>• Vertebrate embryos or tissues<br>• Field research<br><br>Write "N/A" if the submission does not require an ethics statement.<br><br>General guidance is provided below. Consult the submission guidelines for detailed instructions. **Make sure that all information entered here is included in the Methods section of the manuscript.**	Ethical permission to undertake the study was granted by Ethical Committee for College of Basic and Applied Sciences (CBAS), University of Ghana (ECBAS 035/15-16). Permission was also sought from the Ghana Education Service (GES), Municipal and Districts Education Directors in charge of basic schools, community leaders, head teachers, parents and the pupils. Participation in the study was voluntary and subjects could withdraw at any stage of the data collection process without any negative consequences.

Powered by Editorial Manager® and ProduXion Manager® from Aries Systems Corporation

**Format for specific study types**<br><br>**Human Subject Research (involving human participants and/or tissue)**<br>• Give the name of the institutional review board or ethics committee that approved the study<br>• Include the approval number and/or a statement indicating approval of this research<br>• Indicate the form of consent obtained (written/oral) or the reason that consent was not obtained (e.g. the data were analyzed anonymously)<br><br>**Animal Research (involving vertebrate animals, embryos or tissues)**<br>• Provide the name of the Institutional Animal Care and Use Committee (IACUC) or other relevant ethics board that reviewed the study protocol, and indicate whether they approved this research or granted a formal waiver of ethical approval<br>• Include an approval number if one was obtained<br>• If the study involved *non-human primates*, add *additional details* about animal welfare and steps taken to ameliorate suffering<br>• If anesthesia, euthanasia, or any kind of animal sacrifice is part of the study, include briefly which substances and/or methods were applied<br><br>**Field Research**<br><br>Include the following details if this study involves the collection of plant, animal, or other materials from a natural setting:<br>• Field permit number<br>• Name of the institution or relevant body that granted permission	
**Data Availability**<br><br>Authors are required to make all data underlying the findings described fully available, without restriction, and from the time of publication. PLOS allows rare exceptions to address legal and ethical concerns. See the PLOS Data Policy and FAQ for detailed information.	Yes - all data are fully available without restriction

A Data Availability Statement describing where the data can be found is required at submission. Your answers to this question constitute the Data Availability Statement and **will be published in the article**, if accepted.<br><br>**Important:** Stating 'data available on request from the author' is not sufficient. If your data are only available upon request, select 'No' for the first question and explain your exceptional situation in the text box.<br><br>Do the authors confirm that all data underlying the findings described in their manuscript are fully available without restriction?	
**Describe where the data may be found in full sentences. If you are copying our sample text, replace any instances of XXX with the appropriate details.**<br><br>• If the data are **held or will be held in a public repository**, include URLs, accession numbers or DOIs. If this information will only be available after acceptance, indicate this by ticking the box below. For example: *All XXX files are available from the XXX database (accession number(s) XXX, XXX.*).<br>• If the data are all contained **within the manuscript and/or Supporting Information files**, enter the following: *All relevant data are within the manuscript and its Supporting Information files.*<br>• If neither of these applies but you are able to provide **details of access elsewhere**, with or without limitations, please do so. For example:<br><br>*Data cannot be shared publicly because of [XXX]. Data are available from the XXX Institutional Data Access / Ethics Committee (contact via XXX) for researchers who meet the criteria for access to confidential data.*<br><br>*The data underlying the results presented in the study are available from (include the name of the third party*	Data is added as supporting information

and contact information or URL).<br>• This text is appropriate if the data are owned by a third party and authors do not have permission to share the data.<br><br>* typeset	
Additional data availability information:	Tick here if the URLs/accession numbers/DOIs will be available only after acceptance of the manuscript for publication so that we can ensure their inclusion before publication.

Powered by Editorial Manager® and ProduXion Manager® from Aries Systems Corporation

Manuscript

Click here to download Manuscript Culture and OD Dzidzo & Savi 2020 - v1 final.docx

The Influence of Culture on Open Defaecation in Selected Basic Schools in Ghana

Saviour Adjibolosoo[1], Benjamin D. Ofori[1] , Philip B. Adongo[2] , Stephen Afranie[3] & Dzidzo Yirenya-Tawiah[1]*.

[1]Institute For Environment and Sanitation Studies (IESS), University of Ghana, Legon.
[2] School of Public Health, University of Ghana, Legon.
[3]Social Work, University of Ghana, Legon.

*Corresponding Author –Dzidzo Yirenya-Tawiah – dzidzoy@staff.ug.edu.gh

Abstract

Open defaecation remains major public and environmental health concerns and has attracted global attention. This study explored cultural drivers influencing open defaecation behaviour among basic school pupils in the Eastern and Volta Regions all within Southern Ghana. Self-reported data were collected from 400 pupils using structured questionnaire and focus group discussions held. The results of the study indicated that taboos, superstitions and traditional norms in their pooled state explained 63 and 23 percent of variance in pupils' open defaecation intention and actual open defaecation behaviour respectively. In their individual state, these cultural variables yielded statistically significant effect sizes with pupils' open defaecation behaviours: taboos: $r = 0.462$, $p<0.05$; traditional norms: $r = 0.425$, $p<0.05$; and superstition: $r = 0.728$, $p< 0.05$). Superstitions therefore constituted the key cultural driver influencing pupils' open defaecation behaviours. It is recommended that Ministry of Education should integrate the local cultural beliefs systems relating to sanitation practices into the basic school curriculum and ensure that science teaching help pupils give scientific explanations to these cultural beliefs systems that downplay best sanitary practices in the schools. The Ministries of Education and Sanitation and Water Resources should incorporate OD issues into the educational curriculum and develop cultural sensitive educational programs for a massive educational campaign to stop OD in the schools and society at large.

Keywords: Sanitation; open defaecation; behaviour; education; sustainable development goals; Ghana.

1. Introduction

Sanitation is a major cross cutting issue that links 12 out of the 17 Sustainable Development Goals (SDG) giving it prominence on the global agenda. Worldwide, 4.2 billion people live without safely managed sanitation, 673 million still practice open defecation **[1]** . This sanitation crisis means untreated human waste is spreading diseases into water supplies and the food chain for billions of people **[2]**. Inadequate sanitation is estimated to cause 432,000 diarrhoeal deaths every year **[2].**
According to a 2017 report of the Joint Monitoring Programme (JMP) of the United Nations International Children's Emergency Fund (UNICEF) and the World Health Organization (WHO), 31.9 per cent population in the Sub-Saharan Africa practices open defecation and around 62.7 per cent still lacks any facilities of hygiene **[3].**

Globally 23% of schools have no sanitation service at all and fewer than 50% of schools have toilets accessible to students with limited mobility **[4]**. Unfortunately, sanitation coverage in schools is lowest in sub Saharan Africa **[3]** in general although there are country level variations. Tanzania reported the lowest

coverage of toilets in schools (11%), Niger (14%) and Congo (15%). South Africa had the highest coverage of (100 %), Rwanda (95%) and Ghana, (62%) **[3].**

Previous reports had shown that inadequate water and sanitation facilities in school environment was a major hindrance towards achievement of the MDGs **[4]** and will continue to hinder the achievements of SDGs if countries do not invest in improving the sanitation. **[5]** observed that, even in places with high toilet coverage levels, open defecation is still practiced. They therefore suggest that user's decision to openly defecate were likely to be influenced by a number of technological and behavioral factors **[6] [7] [8]**. In some areas of India and East Java for instance, open defaecation is a social norm and is strongly nurtured by allocation of sites for it **[9]**. Other studies also revealed that in some ethnic cultures, traditional beliefs such as a father-in-law and a daughter-in-law cannot use the same toilet and hence compelled many people to go for open defaecation options **[10].** In other places, menstruating women are banned from toilet use with a belief that they are untouchable during those menstruating days **[11].**

In West Africa, the practice of open defaecation is also surrounded by cultural taboos and beliefs **[12] [13]**. OD in the region is found to be related to factors specific to the culture of the ethno-linguistic group that practices it. **[12]** noted that fear of being possessed by demons and losing one's magical powers was a leading cause of open defaecation behaviour seen across all the study areas. The impact of social norms on safe excreta disposal has also been identified by **[13]**. They found people imitate others (defaecating indiscriminately) thinking that it is good practice. This paper reports aspects of a larger study that sought to understand behavioural factors that influence open defaecation behaviour.

Existing evidence show that placing emphasis on provision of hard ware sanitation facilities does not necessarily result in improved sanitation. **[14]** in their systematic review of literature on how different sanitation interventions impact latrine coverage and use, found from their study that, most sanitation interventions only had a modest impact on increasing latrine coverage and use. They recommended further understanding of how different sanitation characteristics and sanitation interventions impact coverage and use if open defecation is to be eliminated, and ultimately improve health.

This paper reports aspects of a larger study that sought to understand behavioural factors that influence OD behaviour. Behavioural factors that influence toilet use have not been widely studied within school settings as compared to community and household levels. The need for understanding cultural reasons why people defaecate in the open, even though they have toilet facilities, can contribute significantly to development of culturally appropriate measures that will help shift such open defaecators to sustainable toilet use.

2. Materials and Methods

2.1 Conceptual Framework

The conceptual framework guiding the conduct of this study is adapted from Ashebir *et al* (2013) **[15].** (Figure 1). This framework postulates that OD practice is influenced by intrinsic individual behaviour which depends on attitude, subjective norms and perceived behavioural control **[16],** and and other external factors such as the availability or otherwise of physical facility. Literature is replete on the influence from the behavioural context on sanitation. However, the role of the various variables that make up behaviour (e.g. culture) have not been extensively studied.

Figure 1. Authors construct (2017)

2.2 Study Area and Communities

The study was conducted in two selected districts in Volta and Eastern regions, both of which are located in Southern Ghana (Figure 2) These districts were purposively chosen based on previous reports of high open defaecation prevalence rates **[17]**. The Eastern Region districts are; Fanteakwa located on longitudes 0°32.5' West and 0°10' East and latitudes 6° 15' North and 6° 40' North and Nsawam Adoagyiri - latitude 5'.45 N and 5'.58 N and longitude 0.07'W and 0.27' W. The residents are predominantly Akans and mainly engaged in farming (Field observation, 2016). The Volta Region districts studied are Akatsi South- located between latitude 6° S - 7° North 0° W - 1 ° W and Keta- Longitudes 0.30E and 1.05E and Latitudes 5.45N and 6.005N. Majority of the inhabitants from Keta and Akatsi are fishers, farmers, traders. Two communities were selected from each district and one basic school selected from each community making a total of eight basic schools studied. All the study communities had at least one basic school with a functioning toilet facility.

All the study communities relied mainly on streams, rivers, bore holes, and wells for their source of water for domestic use. None of the schools studied had a water system on its compound. The common sanitation facility used in these schools are the Kumasi Improved Ventilated Pit (KVIP) latrines. The average enrolments of selected study schools in Nsawam and Fanteakwa was 248 and Keta and Akatsi South was 308.

Figure 2: Map Showing Study Districts and Schools

2.2 Data Collection

The cross-sectional study design was employed to understand cultural factors that influence OD among pupils in selected basic schools. The primary measure of open defaecation was reported as practice of open defaecation in past one week prior to the survey. The one week recall was based on the premise that the prevailing environmental situation in the study areas will have been the same as the previous one week. A mixed method approach comprising both quantitative and qualitative instruments were employed. The selected variables that were assessed for their influence on OD practice were based on literature, expert's opinion and also drawn from interactions with the study community members during reconnaissance visits. The cultural variables listed below were categorized as superstition, taboos, traditional norms and traditional value (Table 1).

Table 1: Categorization of Cultural Variables

Variables	Representation	Categorization
Superstition	[S1]	Defecating in toilet causes one to be possessed by evil spirits.
	[S2]	Shared toilet are associated with evil spirits and therefore should avoided.
	[S3]	Using same toilet with people in the other houses causes one to lose his/her social status.
	[S4]	Defecating in public toilet causes one to lose his/her magical powers.

**Traditional norms**	**[T1]**	Faeces are not things to be kept in homes/ in toilet in the house.
	**[T2]**	Girl who are in their menstrual periods are not supposed to defecate in toilet.
	**[T3]**	Children can defecate in the open since their feces are considered harmless.
**Traditional value**	**[TV1]**	Defecating in the open (bush, beaches) signifies continuation of ancestor's wat of life.
	**[TV2]**	Contact with human faeces is unacceptable.
**Taboo**	**[TB]**	Defecating in an enclosed place (inside toilet) is a taboo).

Quantitative data was gathered using a pre tested structured questionnaire which was tested using the Cronbach's Alpha reliability test for the internal consistency of the instrument. Qualitative data was collected through focused group discussions and interviews. The instruments captured data on demographics, knowledge awareness, knowledge and perception of open defaecation (published elsewhere) and cultural issues.

A total of 400 basic school pupils between Classes 4 to Junior High School (JHS) form 3 (equivalent of Grades 4 to 9) were enrolled into the study and [illegible]5 teachers interviewed to validate information provided by the pupils. Prior to the data collection two Research Assistants were recruited and trained on study procedures as well as translation of concepts for the appreciation of the pupils in both English and main local dialects spoken in the study communities. Although the survey was administered mainly in English, periodically, the local dialects (such Twi and Ewe) were used to clarify information being communicated when required.

Ethical permission to undertake the study was granted by Ethical Committee for College of Basic and Applied Sciences (CBAS), University of Ghana (ECBAS 035/15-16). Permission was also sought from the Ghana Education Service (GES), Municipal and Districts Education Directors in charge of basic schools, community leaders, head teachers, parents and the pupils. Participation in the study was voluntary and subjects could withdraw at any stage of the data collection process without any negative consequences.

2.3 Data Analysis

The data collected was cleaned and checked for completeness and consistencies and entered into the SPSS software version 20 (SPSS Inc. Chicago, IIlinois, USA). The Cronbach's alpha reliability test was conducted to test the internal consistency of the instrument and goodness of fit of each model. These were used to determine the cultural influences on OD behaviour. Chi-square test was used to determine association between the demographics and cultural variables that influence OD the variables that were significant were then further analysed with the AMOS software version 24. The AMOS was development to satisfy the data process demand from research effort. One major importance problem in the data gathering instrument was the extent to which the measurement scale does measure that

which they were supposed to measure, in other words, the degree to which their factorial structures were valid. One of the most strictly methodological approaches to ensure for the validity of dimensions structures was the use of confirmatory factor analysis (CFA) within the framework of structural equation modeling. Structural Equation Model (SEM) was then applied to the data to estimate the relative mean moderating effect sizes of cultural variables (taboos, superstition, traditional norms and traditional values) on intention to open defaecate and the practice of open defaecation dependent variables (Figure 3).

The effect of the pooled cultural variables were also assessed. The measurement scale of the effect size is within the range of 0-1. Negative effect sizes are considered not to influence OD behaviour. All the analysis was conducted at 95% significant levels.

Figure 3.Path Diagram for Estimating Moderating Effect Size of Individual Cultural Variables on Pupils Open Defaecation Behaviour through intention *(M_2)*.

3.0 Results

3.1 Findings from Quantitative Assessment

3.1.1 Demographic Characteristics of study participants

The demographic profile of the study participants are presented in Table 2. Significant variations were observed between the characteristics studied in the selected districts. In Fanteakwa and Nsawam, there were more males (54.0% and 60.0% respectively) than females participants whereas Akatsi had more females (64.0%) than males (36.0%) while Keta had equal representation of females and males. The mean age of the study participants was 15.7 years and majority of the pupils were in JHS. Most of participants were Christians and spoke the ewe language.

Table 2. Demographic Profile of Study Participants

	**Eastern Region**		**Volta Region**		**Total**
	**Fanteakwa**	**Nsawam-Adoagyiri**	**Akatsi**	**Keta**	
**Characteristic**	**Respondents (%)**	**Respondents (%)**	**Respondents (%)**	**Respondents (%)**	**Respondents (%)**
**Sex**					
Male	54(54.0)	60 (60.0)	36 (36.0)	50 (50.0)	200 (50)
Female	46 (46.0)	40 (40.0)	64 (64.0)	50 (50.0)	200 (50)
Chi square (p-value) 19.7 (0.003)					
**Age Group**					
9-11	12 (12.0)	7 (7.0)	20 (20.0)	20(20.0)	59 (14.8)
12-15	39 (39.0)	48 (48.0)	55 (55.5))	28 (28.0)	200 (50.0)
16-18	49 (49.0)	45(45.0)	25 (25.0)	22 (22.0)	41 (35.2)
Mean age.15.7; Chi-square (p-value): 44.1 (0.830)					
**Educational level**					
P4-P6	45 (45.0)	51 (51.0)	36 (36.0)	60 (60.0)	192 (48.1)
JHS 1- JHS 3	55 (55.0)	49 (49.9)	64 (64.0)	39 (39.0)	207 (51.9)
Chi-square(pvalue) 43.8 (0.173)					
**Religion**					

Christian	92(92.0)	88(88.0)	88(88.0)	88(88.0)	
Islam	5(5.0)	10(10.0)	9(9.0)	12(12.0)	--
Traditional	3 (3.0)	2(2.0)	3 (3.0)	0(0.0)	
Chi square (p-value) 35.8 (0.602)					
**Ethnicity**					
Ga	6(6.0)	22(22.0)	6(6.0)	5(5.0)	39 (9.8)
Ewe	52(52.0)	28(28.0)	55(55.0)	81(81.0)	216 (54.0)
Akan	28(28.0)	47(47.0)	38(38.0)	9(9.0)	122 (30.5)
Ga dangme	12(12.0)	3(3.0)	0(0.0)	1(1.0)	16 (4.0)
Others	2(2.0)	0(0.0)	1(1.0)	4(4.0)	17 (1.8)
Chi square (p-value) 20.1 (0.025)					

3.1.2 Pupils Responses to Various Cultural Variables Tested

Tables 3 to 6 present the frequency of responses by pupils regarding selected key questions used to test for the cultural variables on a liket scale. Generally, majority of the pupils either strongly disagreed and/or disagreed that the cultural variables tested informed open defaecation behaviour. However, it was also observed that quite a number of the respondents, about 10-30% agreed and/or strongly agreed to cultural variables informing OD behaviour. It was generally observed that more males than females agreed to the knowledge that shared toilets is associated with evil spirit and so must be avoided and that, girls in their menstrual periods are not to defaecate in toilets. Also, it was observed that the younger pupils (9-13 years) generally agreed to tested variables compared to the older pupils.

Table 3: Pupils Responses to Traditional Norms/Beliefs about Open Defaecation

**Question 1: Defaecating outside toilet signifies continuation of ancestral ways of life**						
**Percentage (%) Distribution of Choice Categories in the Sample**						
**Study Schools From:**	Strongly disagree	Disagree	Don't Know	Agree	Strongly agree	
**Districts**						
Fanteakwa	54	16	8	14	8	100
Akatsi	48	22	5	13	12	100
Keta	55	18	2	17	8	100
Nsawam-Adoagyiri	54	23	12	5	6	100
**Locality**						
Rural	44 (22.0)	93 (46.5)	16(8.0)	36(18.0)	11(5.5)	200(100)
Urban	77 (38.5)	59 (29.5)	30(15.5)	26(18.0)	8(4.0)	200(100)
**Sex**						
Male	28(14.0)	96(48.0)	21(10.5)	43(21.5)	12(6.0)	200(100)
Female	19(9.5)	112(56.0)	33(16.5)	28(14.0)	8(4.0)	200(100)
**Age Group(Year)**						
9-13	100(54.6)	32(17.5)	8(4.4)	26(14.2)	17(9.3)	183(100)
14-18	111(51.2)	47(21.7)	19(8.8)	25(10.6)	15(9.3)	217(100)

Table 4: Pupils Responses to Superstitions about Open Defaecation

**Shared toilets are associated with evil spirits and therefore should be avoided**						
**Frequency/Percentage Distribution of Choice Category in the Sample**						
**Study Schools From:**	Strongly disagree	Disagree	Don't Know	Agree	Strongly agree	
**Districts**						
Fanteakwa	58	24	11	5	2	100
Akatsi	36	35	18	7	4	100
Keta	68	14	5	13	0	100
Nsawam-Adoagyiri	44	26	12	10	8	100
**Locality**						
Rural	119	25	13	32	11	200
Urban	92	31	17	41	19	200
**Sex**						
Male	89 (57.0)	57(23.5)	15 (7.5)	28(14.0)	11(5.5)	200
Female	111(55.5)	43(21.5)	14(7.0)	19(9.5)	13(6.5)	200
**Age group (Year)**						
9-13	92(50.3)	31(16.9)	22(12.0)	32(17.5)	6(3.3)	183
14-18	114(52.5)	58(26.7)	24(11.1)	13(6.0)	8(3.7)	217

Table 5: Pupils Responses Knowledge about Taboo on Open Defaecation

Defaecating in an enclosed place (inside toilet) is a taboo						
	Frequency/Percentage Distribution of Choice Category in the Sample					
**Study Schools From**	Strongly disagree	Disagree	Don't Know	Agree	Strongly agree	% Distribution
**Districts**						
Fanteakwa	68	27	1	3	1	100
Akatsi	45	25	10	14	5	100
Keta	74	9	2	9	6	100
Nsawam-Adoagyiri	55	27	10	4	4	100
**Locality**						
Rural	42(21.0)	109(54.5)	18(9.0)	22(11.5)	9(4.5)	200
Urban	32(16.0)	123(61.5)	9(4.5)	17(8.5)	19(9.5)	200
**Sex**						
Male	40 (20.0)	97(48.5)	15(7.5)	28(14.0)	20(10.0)	200
Female	16 (8.0)	112(56.0)	32(16.0)	24(12.0)	16(8.0)	200
**Age Groups(Year)**						
9-13	104(56.8)	43(23.5)	7(3.8)	19(10.4)	10(5.5)	183(100)
14-18	138(63.6)	45(20.7)	16(7.4)	12(5.5)	6(2.8)	217(100)

Table 6: Pupils Responses to Traditional Values Regarding Open Defaecation

Girls who are in their menstrual periods are not supposed to defaecate in toilets						
	Frequency/Percentage Distribution of Choice Category in the Sample					
**Study Area:**	Strongly disagree	Disagree	Don't Know	Agree	Strongly agree	
**Districts**						
Fanteakwa District	60	15	13	4	8	100
Akatsi South District	45	36	9	6	4	100
Keta Municipal	70	17	7	4	2	100
Nsawam-Adoagyiri Municipal	51	21	17	6	5	100
**Locality**						
Rural	21(10.5)	98(49.0)	22(11.0)	44(22.0)	15(7.5)	200
Urban	71(35,5)	63(31.5)	13(6.5)	32(16.0)	21(10.5)	200
**Sex**						
Male	98(49.0)	23(11.5)	15 (7.5)	34(17.0)	19(9.5)	200
Female	117(58.5)	39(19.5)	18(9.0)	13(6.5)	7(3.3)	200
**Age Groups (Year)**						
9-13	86(47.0)	29(15.8)	22(12.0)	23(12.6)	23(12.6)	183
14-18	123(56.7)	74(34.1)	18 (8.3)	21(9.7)	2(0.1)	217

Table 7: Association Between Demographic and Culture and Open Defaecation Practice

**Independent Variables**	**Open Defaecation Intention**		**Open Defaecation Behaviour**		**Cronbach's Alpha**
	χ^2	**p-values**	χ^2 **Values**	**p-values**	
**Pupils Demographic Characteristics**					.85
Sex	1.264	0.867	19.671	**0.003***	
Settings	4.394	0.355	20.087	**0.003***	
Age	17.678	0.798	44.082	0.830	
Education	17.701	0.818	43.841	0.173	
Ethnicity	39.067	0.334	20.087	**0.025***	
Religion	5.646	0.687	10.158	0.602	
**Cultural Factors**					
T1	76.240	0.00*	39.398	0.00*	
T2	25.109	0.068	40.431	0.02*	
T3	36.387	0.003	33.560	0.09	
TV1	38.398	0.001	…..	….	
TV2	42.995	0.000	37.919	0.035	
TB	39.572	0.006	39.370	0.12	
S1	31.684	0.01*	34.289	0.08	
S2	23.152	0.11	27.223	0.29	
S3	30.540	0.015*	60.422	0.00*	
S4	62.010	0.00*	27.122	0.299	

**Independent variables which show significant association with dependent variables (open defaecation intention & open defaecation behaviour. Correlation is significant at 0.05 level (two-tailed) and 0.001 level (one-tailed).*

3.1.3 Association Between Demographic and Culture and Open Defaecation Practice

Table 7 presents findings from the initial assessment aimed at exploring association between the demographic variables and OD as well as culture and OD among the pupils. OD was assessed based on intention and actual practice of the behaviour. The study revealed none of the demographic variables were associated with the intention to defaecate, whereas sex, setting and ethnicity were the variables associated with OD behaviour. On the other hand, individual cultural factors T1, T3, TV1, TV2, TB, S1 and S3 were found to be associated with intention to OD and also T1, T2, TV2 and S3 were found to be associated with OD.

On the other hand, further assessment of the effects of demography on OD behaviour using the SEM model also did not reveal any effect except for location (Table 8).

Table 8. Moderating Effect Size of Demographic Variables on Pupils Open Defaecation Behaviours

Coefficient Table for Demographic Characteristics on Open Defaecation

Independent Variables	Impact	Dependent Variables	Standardized Estimates	S.E.	t-statistics	P-value
Sex	----->	Open Defecation	-0.028	0.198	-0.566	0.571
Age	----->	Open Defecation	-0.026	0.057	-0.522	0.602
Education	----->	Open Defecation	-0.038	0.046	-0.761	0.447

Location	----->	Open Defecation	0.101	0.188	2.033	0.042*

*All coefficients significant at $p<0.05$

Analysis of the cultural variables using SEM model to establish the strength of the relationship between the cultural variables that were revealed to be associated with OD behaviour among the pupils is shown in table 4. Superstition showed the largest effect size (r = 0.728; p = 0.00) compared to taboos (r = 0.46) and beliefs (r = 0.43). Traditional values showed the least effect (r = 0.28). Likewise, regarding the assessment of cultural values effects on intension to engage in OD, superstition was significant with standardized effect = 0.138; p = 0.005. (Table 9). Taboos on the contrary were found to have an inverse significant effect size with pupils' intention to engage in open defaecation: standardized effect = -0.098, p = 0.048). Traditional values and beliefs on the other hand did not influence pupils' intention to open defaecation.

This result indicates that, pupils have difference cultural reasons for avoiding toilet use or defaecating in the open. This was expressed by discussants during the FGDs.

For example, a discussant from Kofisah M.A basic school said that "*he avoided toilet use because of fear of being possessed by evil spirit*".

Another discussant from Kofisah M.A. Basic School also explained: "*I defaecate in the open because the chief of my community also does it".*

Further, a discussant from Akuffokrom M.A. Basic school also expressed his view as "Defaecating *in the bush is not a taboo because visitors had to go there if the community has no toilet."*

The moderating effect size of pooled cultural variables on pupils open defaecation intention and open defaecation behaviour is presented in Table10. The result shows that, in their pooled state, culture has statistically significant effect size on pupils open defaecation intention and actual open defaecation behaviour.

Table 9. Moderating Effect Size of Demographic Variables on Pupils Open Defaecation Behaviours

SEM Path Model	(a) Effect Size of Individual Cultural Variables on Pupils Open Defaecation Behaviour						
	Independent Variables	Impact	Dependent Variables	Standardized Estimates	S.E.	t-statistics	P-values
M_6	Taboos	---->	Open Defecation	0.462	0.115	33.406	0.00
	Beliefs	----->	Open Defecation	0.425	0.105	41.541	0.00
	Superstition	----->	Open Defecation	0.728	0.180	59.933	0.00
	Tradition (value)	----->	Open Defecation	0.276	0.068	29.972	0.00

Table 10: Pooled Effect Size of Cultural Factors on Pupils' Open Defaecation Intention and Open Defaecation Behaviour

(b) Effect Size of Individual Cultural Variables on Pupils Open Defaecation Intention

	Independent variables	Impact	Dependent Variables	Standardized Estimates	S.E.	t-statistics	pvalues
	Traditional value	----->	Intention	0.012	0.09	0.247	0.805
M_7	Superstition	----->	Intention	0.138	0.034	2.814	0.005*
	Beliefs	----->	Intention	0.077	0.059	1.566	0.117
	Taboos	----->	Intention	-0.098	0.054	-1.98	0.048*

**All coefficients significant at* $p<0.05$

**SEM Path Models**	**Independent Variables**		**Dependent Variables**	**Standardized Effect Size (r)**	**S.E.**	**t-statistics**	**p-value**
**M_3**	Pooled cultural factors	------>	OD Intention	0.63	0.653	9.25	0.000*
	**OD Intention**	----->	OD behaviour	0.23	0.252	3.87	0.021*

4.0 Discussion

Understanding the influence of culture on OD practice is as important as other factors that influence the practice of OD. **[18]** in their study found physical, emotional and social drivers were identified as factors that influence sanitation behaviour. The influence of culture as a social factor has also been suggested in many publications [1]. However not many studies have analysed the various cultural factors and their effect on OD. Our study sought to unravel the effect of culture various cultural variables -on sanitation behaviour among basic school children in some selected districts in Ghana. The study found most of the pupils (>30%) from all studied schools generally disagreed that culture, captured in this study as taboos, superstition, traditional norms and cultural values informed OD behaviour. Although more of the pupils generally disagreed that cultural factors informed OD, there were still pupils who agreed that culture informed OD behaviour. To explore possible association between the demographic characteristics and predictor cultural variables and OD behaviour, it was found that sex, location and ethnicity were associated with OD**,** respectively and not intention to OD.

The fact that location had an influence on OD behaviour maybe as a result of sanitation access issues. Access to sanitation facility may go beyond the provision of the physical infrastructure to include

maintenance issues. Where sanitation blocks for instance are available but not cleaned or in dilapidated situations may also influence its use and OD behaviour. Our study showed most of the rural schools to have less quality toilet facilities than the urban schools.

The SEM model was used to further analysis the extent of influence of demography and predictor cultural variables on OD behaviour. This assessment was guided by the path model which is derived from the Adjen behaviour theory, where behaviour is mediated by intention to perform the behaviour **[16].** Regarding the demographic characteristics, only the location was found to predict OD. We observed more pupils in rural areas agreed to OD behaviour than pupils in urban areas. In the case of the predictor cultural variables assessed, superstition was found by this study to show the strongest influencer of OD behaviour. Many cases of cultural intolerance for the handling of faeces are associated with superstitious beliefs and are common in countries such as China, India and Ghana **[19] [20] [21] [22].** Taboo, however, was found to have an inverse relationship with OD intention suggesting that about 9.8% of the variability of developing intention to engage in OD is inhibited by taboo. **[23]** who found men to refuse to stop open defaecation because of f toilet-associated taboos with in-laws and grown-up children of the opposite sex.

This result is in congruent with a study by **[23]** who found men to refuse to stop open defaecation because of toilet-associated taboos with in-laws and grown-up children of the opposite sex. Traditional norms were generally not significant in influencing OD behaviour in this study. This observation is contrary to many findings such as [24] in Wa in Northern Ghana that found local traditional norms, taboos and beliefs could determine OD practic[illegible]nd **[25]**, who suggest perceived social sanitation norms may enhance emotional satisfaction where OD practice is rampant and negate movement up the sanitation ladder. **[11]** also notes that social norms which were rooted in culture and tradition are learnt as children. It is therefore unclear why norms did not influence OD behaviour in this study and calls for further studies to clarify the findings.

Overall, the results of this study showed culture as a pooled factor to influence OD behaviour in the studied schools. This may be attributed to the fact that behaviour of children leans towards the cultural norms of their families, peers, and communities **[26]**. Throughout existence, an individual behaviour is influenced by his/her environment—family, friends, peers, mass media, school **[26]** and more importantly the cultural environment that teaches him values, preferences as well as common cultural behaviours **[26].** Further, current research on goal pursuit has thoroughly demonstrated that significant others influence individuals' goal-directed behaviours **[27].** Individuals, according to this study, are more likely to engage in goal pursuits of salient others **[27]**.

The general finding from our study that culture influences OD behaviour is also comparable to a study conducted by **[13]** and **[9]** to determine the cultural factors influencing open defaecation behaviour in rural settings in Ghana, Burkina Faso, Mali, and Nigeria, who found that taboos, beliefs and values are major cultural factors influencing open defaecation behaviour among study participants Studies **[28]** in Krishnagiri, Pudukottai and Salem Districts reported that people avoided toilet use because they believed it was unhygienic and culturally wrong to have toilets near their houses. They also reported that using toilets was culturally unacceptable **[28]** and they are perceptually habituated to open defaecation **[28]**. A similar study in Sierra Leone **[29]** also demonstrated that people belief that one should not sit over someone else's faeces because it will lead to bad luck; and an initiated individuals should not sit over the shit of a non-initiate. Reported in another previous study in Kampala also asserted that people believe that pregnant women should not use the toilet because of fear of the death of the foetus **[28].**

To promote the change in sanitation culture and reduce the practice of OD in basic schools in Ghana and contribute to meeting SDG target 2.1, school toilets should be accessible by putting in place a WASH maintenance system by the Ghana Education Service which must be regularly monitored. In addition to this, school management must find innovative ways of maintaining the facilities to meet expected level of cleanliness to facilitate its use. Noting the challenges of resource provision by Ghana Education Services, School management can engage with their community leaders and members, political heads, NGOs and other key stakeholders to support in WASH services for schools.

A review of the Ghana Education Service new curriculum for basic schools does not seem to emphasis issues of open defaecation **[30]**. Unfortunately, 18% of Ghanaians practice OD yet this has not been captured as key issue in the basic school science curriculum. We therefore recommend an update of the revised basic school curriculum to include open defaecation and related social issues in subjects' areas dealing with humans and the environment.

It is also important that, teachers in the first cycle schools must know that the pupils they teach come from different cultural background and can be more or less influenced by parental sanitation behaviours. As a consequence, it is important that teachers collaborate with parents to instill in pupils' positive attitudes that will help them avoid open defaecation behaviour. They must develop sanitation activities that can promote development of proenvironmental sanitation behaviour in the pupils they teach. A study **[31]** suggests that teachers should be "producers of environment that allow pupils to learn good sanitation behaviours as much as possible". Nevertheless, teachers should be provided with training to support them in this expanded role: more time for peer interaction to share views on what is effective when it comes to good school sanitation behaviour promotion.

In conclusion, it is recommended that massive educational efforts should be done by the Ministry of Saniation and Water Resources to promote school sanitation and stop the practice of OD. The design of any such educational program should intricately include cultural dimensions noting that sanitation and hygiene behaviours may be entrenched within long-standing cultural factors. To ensure that messaging resonates target audience, communication campaigns should promote a number of positive emotional and social, drivers, such as improved social status and positive self-esteem with toilet use.

Conflict of Interest
The authors declare no conflicts of interests.

Acknowledgement
The authors are grateful to the staff and pupils of the study schools for their contribution to the success of this study. The authors are equally grateful to Carnegie, University of Ghana Chapter, for their partial financial support.

REFERENCES

[1]WHO/UNICEF. Joint Monitoring Programme 2019 update report: Progress on household drinking water, sanitation and hygiene; 2019.

https://www.who.int/water_sanitation_health/publications/jmp-report-2019/en/

[2] World Health Organization and UNICEF . Progress on sanitation and drinking water 2013 update. World Health Organization and UNICEF; 2013.

[3] World Health Organization and UNICEF. Progress on sanitation and drinking-water-

2019 update. World Health Organization and UNICEF; 2019.

[4] UNICEF and WHO.Drinking water, sanitation and hygiene in schools Global baseline report . New York: United Nations Children's Fund (UNICEF) and World Health Organization; 2018. License: CC BY-NC-SA 3.0 IGO.

[3] Sengupta, S. "Ethiopia curbed open defecation at fastest rate; what can India learn?" Down to Earth, New Delhi. Available at http://www.downtoearth.org.in/news/ethiopia-pushes-hygiene-57463, as accessed on 2 January 2018 S; 2018.

[4] Jasper C. Le T.-T. Bartram J.Water and sanitation in schools: a systematic review of the health and educational outcomes . Int J Environ Res Public Health. 2012 Aug;9(8): 2772-87. doi: 10.3390/ijerph9082772. Epub 2012 Aug 3.[cited 2016 February 19]. Available from: https://www.ncbi.nlm.nih.gov/pubmed/23066396.

[5] Barnard S., Routray P., Majorin F., Peletz R., Boisson S., Sinha A., Clasen T. Impact of Indian Total Sanitation Campaign on latrine coverage and use: a cross-sectional study in Orissa three years following programme implementation. PLoS One. 2013; 8:e71438. [PMC free article] [PubMed] [Google Scholar].

[6] Coffey, D., A., Gupta. Hathi, N., Khurana, D., Spears, N., Srivastav, & S., Vyas. Revealed preference for P open defecation: Evidence from new survey data. *Economic & Political Weekly. 2014; 49*(38): 43-55. [cited 23 March 2017]. Avaiable from

https://www.researchgate.net/publication/286771583_Revealed_preference_for_open_defecation_Evidence_from_a_new_survey_in_rural_north_India.

[7] Hulland, K., Martin, N., Dreibelbis, R., DeBruicker, V., Winch, P., 2015. What factors affect sustained adoption of safe water, hygiene and sanitation technologies? 3ie Systematic Review Summary 2. International Initiative for Impact Evaluation (3ie), London. Google Scholar hygiene practices—Case study in the Northern Province. *Water Sci. Technol*. 2001; 44: 109–117.

[8] Routray P., Schmidt W.P., Boisson S., Clasen T., Jenkins M.W. Socio-cultural and behavioural factors constraining latrine adoption in rural coastal Odisha: an exploratory qualitative study. *BMC Public Health*. 2015;15:880. [PMC free article] [PubMed] [Google Scholar]

[9]Water and Sanitation Program (WSP). *Ghana Country Profile: Economic Impacts of Poor Sanitation in Africa. WSP. 2012.*

[9] Water and Sanitation Program (WSP), IFC (International Finance Corporation), & Ministry of Health, Kenya. "Kenya Onsite Sanitation: Market Intelligence." Nairobi, Kenya: Water and Sanitation Program, World Bank. 2013.

[10] Kathryn, O. What Influences Open Defecation and Latrine Ownership in Rural Households? Findings from a Global Review, Washington, DC 20433. 2014; [cited 2015 December Available online @worldbank.org or visit www.wsp.org. .

[11] O'Connell, K. *What Influences Open Defecation and Latrine Ownership in Rural Households*? Findings from a Global Review, Washington, DC 20433. 2014. Available online @worldbank.org or visit www.wsp.org.Accessed on December 27, 2015.

[12] Dittmer, A. Towards total sanitation: socio- cultural barriers and triggers to total sanitation in West Africa. 2009.

[13] Water Aid (WA). *The Socio-economic Impacts of the Advancing Sustainable Health Initiative* (ASEH) in Bangladesh. Water Aid (WA). 2006.

[13] Water Aid. Abandoning open defecation: Comparism & Adaption of social change dynamics. 2018.Retrieved from www.wateraid.org/documents/social_transformation_study_briefing_note.pdt. 11/October 2019.

[13] Devine, J. Tippy-taps: The role of enabling products in scaling up and sustaining hand washing. *Waterlines.2010; 29:*304–314. [cited 2016 January 12]. Available from: https://es.ircwash.org/sites/default/files/Devine-2010-Beyond.pdf

[14] Garn JV, Sclar GD, Freeman MC, Penakalapati G, Alexander KT, Brooks P, Rehfuess EA, Boisson S, Medlicott KO, Clasen TF. The impact of sanitation interventions on latrine coverage and latrine use: A systematic review and meta-analysis. *Int J Hyg Environ Health*. 2017; 220(2Pt B):329-340. [cited 8 August 2018]. Avaiable from https://www.ncbi.nlm.nih.gov/pubmed/27825597

[15] Ashebir Y, Rai Sharma H, Alemu K, Kebede G. Latrine use among rural households in northern Ethiopia: a case study in Hawzien district, Tigray. *International Journal of Environmental Studies.2013; 70(4):*629–636. [cited 5 June 2016]. Avaiable from https://www.tandfonline.com/doi/abs/10.1080/00207233.2013.835533.

[16] Ajzen, I., & Fishbein, M. The influence of attitudes on behavior. In D. Albarracin, B. T. Johnson & M. P. Zanna (Eds.), *The handbook of attitudes*. 2005; 173–221). Mahwah, NJ: Erlbaum. [cited 29 September 2015]. Available from https://psycnet.apa.org/record/2005-04648-005

[16] Ajzen, I. Attitude structure and behavior. In A. R. Pratkanis, S. J. Beckler, & A. G. Greenwald (Eds.), *Attitude structure and function*. 1989; 241-274. Hillsdale, NJ: Lawrence Erlbaum. University of Ghana http://ugspace.ug.edu.gh 216 ,1989.

[17] Ajzen, I. "The Theory of Planned Behavior," Organizational Behaviour and Human Decision Processes.1991; (50): 179-211. [cited 19 December 2016]. Available from https://www.sciencedirect.com/science/article/pii/074959789190020T

[18] Ajzen, I. . Nature and Operation of Attitudes. *Annual Review of Psychology*.2001; 52: 27–58. [cited 17 April 20117]. Available from http://dx.doi.org/10.1146/annurev.psych.52.1.27.

[17]Water and Sanitation Sector Monitoring Platform (WSMP). *Shared latrine facilities – safe or unsafe? 2015. [cited 25 September 2018].* Available from http://documents.worldbank.org/curated/en/438411468281086437/pdf/7241000REPLACE0x00PUBLIC00CSO0Ghana.pdf

[18]O'Reilly, C.E.; Freeman, M.C.; Ravani, M.; Migele, J.; Mwaki, A.; Ayalo, M.; Ombeki, S.; Hoekstra, R.M.; Quick, R. . The impact of a school-based safe water and hygiene programme on knowledge and practices of students and their parents: Nyanza province, Western Kenya,. *Epidemiol. Infect.* 2008; 136: 80–91. [cited 20 July 2017]. Available from https://www.ncbi.nlm.nih.gov/pubmed/17306051.

[19]Van der Geest, S. Akan Shit: Getting Rid of Dirt in Ghana. *Anthropology Today 14 (3)*, 8-12.1998

[20] Ramaswamy, G.*India Stinking*: Manual Scavengers in Andhra Pradesh and their Work. Chennai: Navayana. 2005.

[21] Esrey, S. A., Gough, J., Rapapor, D., Sawyer, R., Simpson-Hebert, M., & Vargas, J. *Ecological Sanitation*. 1998; Stockholm: Swedish International Development Cooperation Agency. Ghana:[citedNovember,2016].Availablefrom http://wsmpghana.wordpress.com/?s=share+sanitation+safe+or+unsafe.

[22] Hart-Davis, A.. Thunder, Flush and Thomas Crapper: *An Encyclopedia*. London: Michael O' Mara Books. 2008.

[23] Thys, S., Mwape *et al.*,. "Why Latrines Are Not Used: Communities' Perceptions and Practices Regarding Latrines in a Taenia solium Endemic Rural Area in Eastern Zambia", *PLoS, special issue on Neglected Tropical Disease*. 2015; 9(3): Available at http://journals.plos.org/plosntds/article?id=10.1371/journal.pntd.0003570. [cited 10 June 2016].

[24] Osumanu, I.K., Kosoe, E.A., & Ategeeng, F. Determinants of Open Defecation in the Wa Municipality of Ghana: Empirical Findings Highlighting Sociocultural and Economic Dynamics among Households. *Journal of Environmental and Public Health.2019;*Article ID 3075840 | 10 pages | https://doi.org/10.1155/2019/3075840.

[25] Novotný, J., Kolomazníková, J. & Humňalová, H. The Role of Perceived Social Norms in Rural Sanitation: An Explorative Study from Infrastructure-Restricted Settings of South Ethiopia. *Int J Environ Res Public Health. 2017;* 14(7):794. Published online 2017 Jul 17.doi: 10.3390/ijerph14070794.

[26] Berger, K. S., & Thompson, R. A. *The developing person through the life span.* New York: Worth Publishers.1998.

[27] Aarts, H., Gollwitzer, P. M., & Hassin, R. R. Goal contagion: Perceiving is for pursuing. *Journal of Personality and Social Psychology. 2004;* 87: 23-37. [cited 11 February 204]. Available at https://psycnet.apa.org/record/2004-15934-002 https://psycnet.apa.org/record/2004-15934-002.

[28] World Bank. *Economic Impacts of Poor Sanitation in Africa*. 2010. Available online at www.wsp.org.

[29] Mehta, L., & Bongartz, P. 'Community-led total sanitation (CLTS) across the seas: Experiences from Africa with a special emphasis on Ethiopia', *RiPPLE Working Paper12*. RiPPLE: Addis Ababa.2009.

[29] GSS (Ghana Statistical Service). *Ghana 2010 Population and Housing Census: Summary Report of Final Results*, GSS, Accra, Ghana, 2012.

[31] Senge, Peter M., *et al. The fifth discipline field book: strategies and tools for building a learning organization.* New York: Doubleday.1994.

Figure Click here to download Figure Figure 1-conceptual framework.docx

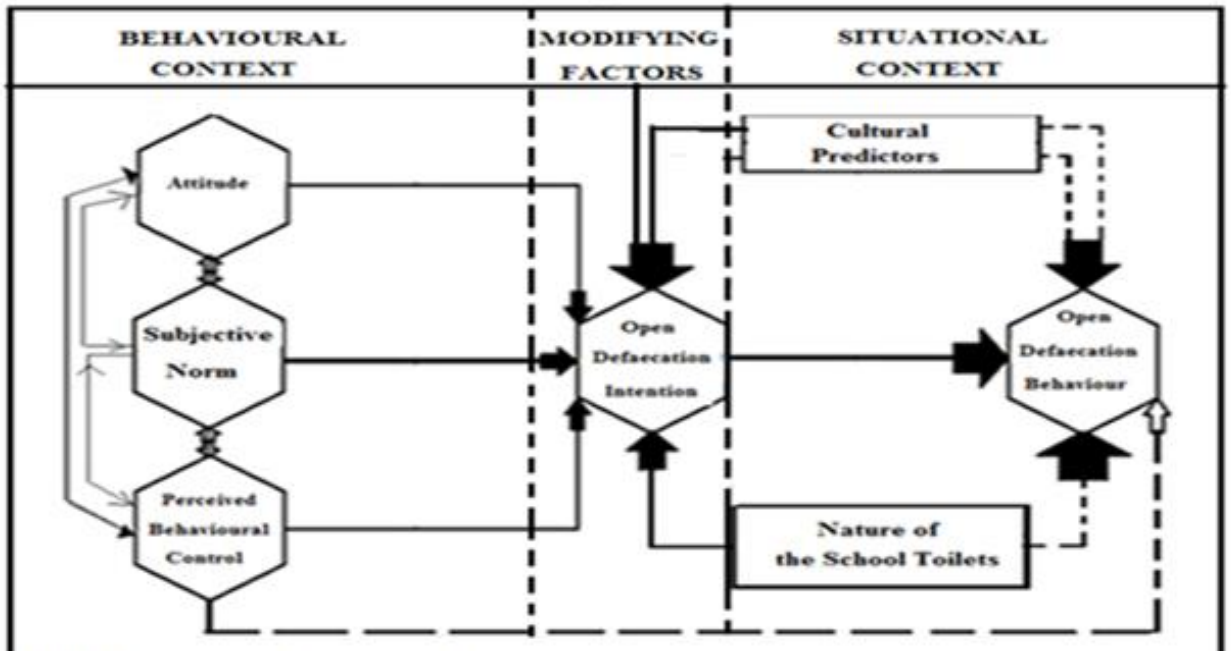

Fig. 1: The Study's Conceptual Framework (Adapted from Barr, 2002b)

Figure 1. Authors construct (2017)

Figure

Click here to download Figure Figure 2-Map of study schools.docx

Figure 2: Map Showing Study Districts and Schools

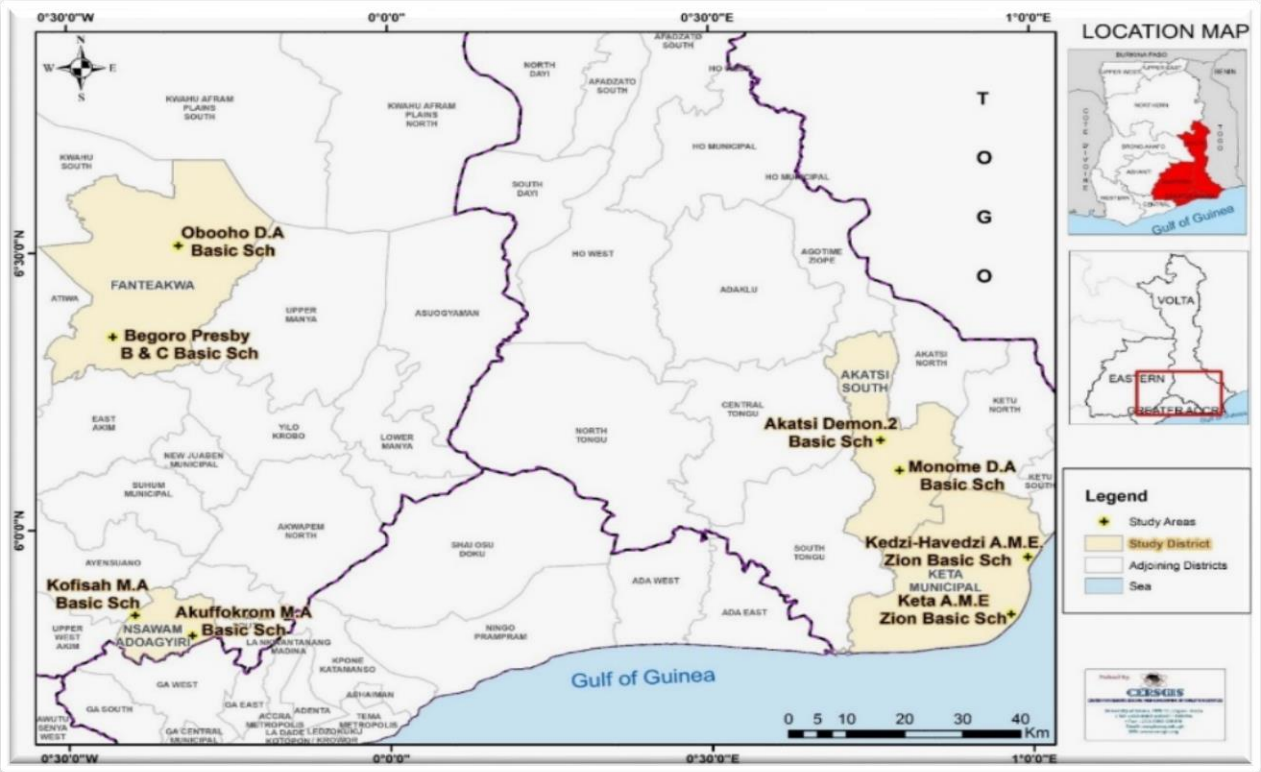

Figure Click here to download Figure Figure 3- path diagram.docx

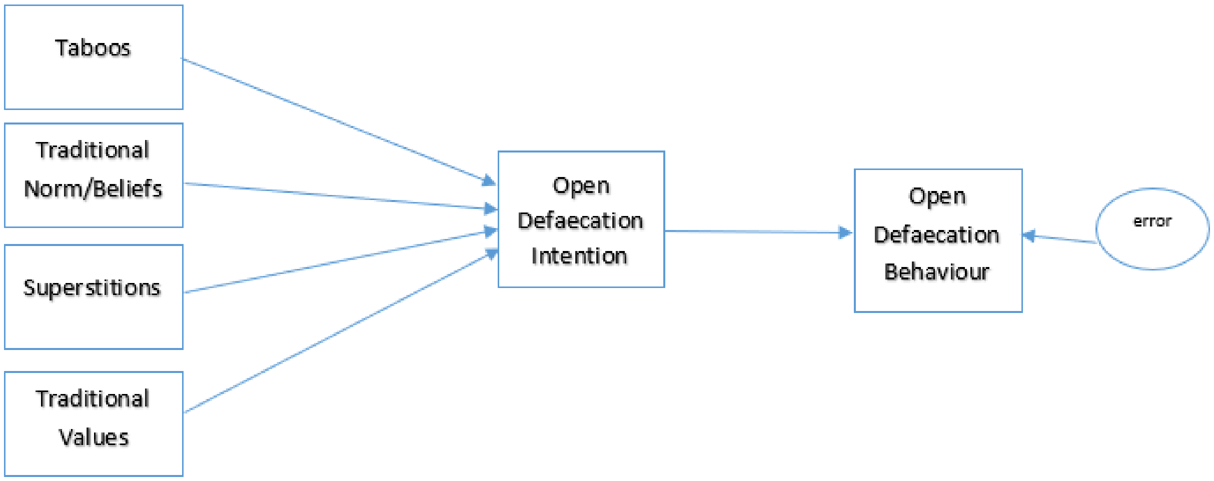

Figure 3: Path Diagram for Estimating Moderating Effect Size of Individual Cultural Variables on Pupils Open Defaecation Behaviour through intention *(M_2). NB. Error indicates other variables that may influence behaviour but not studied*

Click here to access/download

Supporting Information - Compressed/ZIP File Archive

Open defaecation data-saviour.sav1.sav
