# Supplementary material for: The influence of culture on open defaecation in some basic schools in selected districts in Ghana: A preliminary study
Source: PLoS One. 2020 Oct 6;15(10):e0239491. doi: 10.1371/journal.pone.0239491 (PMC7537866; doi:10.1371/journal.pone.0239491)
Supplement: Attachment — Submitted filename: Reviewer comments.docx [file pone.0239491.s002.docx]

**PONE-D-20-14602**

**The Influence of Culture on Open Defecation in Selected Basic Schools in Ghana**

This manuscript is a mixed-methods study describing factors associated with open defaecation among basic school students in Ghana. While the manuscript is of some interest, I do not think the manuscript in its current form is of sufficient interest and quality to warrant publication in PLOS ONE.

I suggest the authors to look for STROBE check list for cross-sectional studies to ensure reporting is complete and transparent.

<https://www.strobe-statement.org/fileadmin/Strobe/uploads/checklists/STROBE_checklist_v4_cross-sectional.pdf>

My sense is that the manuscript could be strengthened by several modest changes as outlined below.

General comments

- The authors should use the uniform font style and font size.
- The manuscript is not well-written and needs to be edited by a native English speaker. Necessary to check grammar and spellings.
- Abbreviate first before using short form.
- Note that writing a manuscript is not the same as writing thesis and it should be built not only with concise and clear statements but also all the required information are included.

INTRODUCTION

- Introduction section is not well organized.
- Some sentences are duplicating [E.g. Line 66 and 77]
- Introduction section needs to include What is the problem and its supporting details? What is the added value from the study and why are you conducting it? - Both globally and locally
- The adverse consequences of open defaecation and its supporting data is missing in the introduction.

METHODS

- The whole section of Methods needs to be rewritten. I would suggest the authors to prepare by the STROBE check list. Many items necessary to be described are missing.
- Sampling procedure: The sampling procedure needs to provide more detail. How schools were chosen in the selected districts? How many students were eligible? Any eligibility criteria? How were they selected? Equal proportion for 4 schools? Gender based selection? Residence based selection?
- Data analysis: It is not clear and should be described in detail. The analysis section should reflect fully to the results that you presented in the study. So also, the way you calculated moderating effect size needs to be described.
- Need to explain qualitative part in more detail: FGDs or Interviews? KII? Any criteria for selection?
- What are the operation definition of “Open defaecation intention” and “Open defaecation behavior”?

RESULTS

- The authors tend to cover all information in the text and again in the table. You should only include the most important details in the text when you also have a table.
- Mean age always come with SD.
- Any reasons for stratification of age groups? Age stratification is different by tables – it should be same
- Percentages are not included in all the data.
- I could not find all your cultural variables S1-4, T1-3, TV1-2 and TB in the results section. I only see one question “Defaecating outside toilet signifies continuation of ancestral ways of life” as traditional norms and same applied to T and TV. Does it a composite measure? And the way you calculated should be described in the Methods section.
- Any reasons behind for cross-tabulating cultural variables with background characteristics?
- There is lacking linkage between qualitative and quantitative results.
- Too many tables some of which can be combined. Figure 1 and 3 may not be necessary.

DISCUSSION

- The discussion is weak in light of the findings. The discussion section should be rewritten. The ideas are incoherently mixed together. The discussion needs to focus on the key implications of the data with a separate paragraph for each concept.
- Check the sentence in discussion “Our study showed most of the rural schools to have less quality toilet facilities than the urban schools.” I do not find it in your results.

REFERENCES

- Number in the references were overlapped and as a result, citations and provided references are not matched.
- Why data from 2013 require when updated 2019 data is available? [Check Ref. 2 and 3 by WHO and UNICEF]
